# Long-Read Sequencing and Hybrid Assembly for Genomic Analysis of Clinical *Brucella melitensis* Isolates

**DOI:** 10.3390/microorganisms10030619

**Published:** 2022-03-14

**Authors:** Hillary A. Craddock, Yair Motro, Bar Zilberman, Boris Khalfin, Svetlana Bardenstein, Jacob Moran-Gilad

**Affiliations:** 1 Microbiology, Advanced Genomics and Infection Control Application Laboratory (MAGICAL) Group, Department of Health Systems Management, School of Public Health, Faculty of Health Sciences, Ben-Gurion University of the Negev, Beer-Sheva 84105, Israel; hcraddock5@gmail.com (H.A.C.); motroy@post.bgu.ac.il (Y.M.); barsch@post.bgu.ac.il (B.Z.); boriskh83@gmail.com (B.K.); 2 Israeli Ministry of Agriculture and Rural Development, Bet Dagan 50250, Israel; svetab@moag.gov.il

**Keywords:** brucellosis, whole-genome sequencing, clinical genomics

## Abstract

*Brucella melitensis* is a key etiological agent of brucellosis and has been increasingly subject to characterization using sequencing methodologies. This study aimed to investigate and compare short-read, long-read, and hybrid assemblies of *B. melitensis*. Eighteen *B. melitensis* isolates from Southern Israel were sequenced using Illumina and the Oxford Nanopore (ONP) MinION, and hybrid assemblies were generated with ONP long reads scaffolded on Illumina short reads. Short reads were assembled with INNUca with SPADes, long reads and hybrid with dragonflye. Abricate with the virulence factor database (VFDB) and in silico PCR (for the genes *BetB*, *BPE275*, *BSPB*, *manA*, *mviN*, *omp19*, *perA*, *PrpA*, *VceC*, and *ureI*) were used for identifying virulence genes, and a total of 61 virulence genes were identified in short-read, long-read, and hybrid assemblies of all 18 isolates. The phylogenetic analysis using long-read assemblies revealed several inconsistencies in cluster assignment as compared to using hybrid and short-read assemblies. Overall, hybrid assembly provided the most comprehensive data, and stand-alone short-read sequencing provided comparable data to stand-alone long-read sequencing regarding virulence genes. For genomic epidemiology studies, stand-alone ONP sequencing may require further refinement in order to be useful in endemic settings.

## 1. Introduction

Microbial genomics analysis is widely being recognized as a potentially useful method to diagnose difficult-to-detect organisms and provide real-time surveillance for outbreaks [1,2]. However, traditional short-read sequencing methodologies have drawbacks in terms of contig length and turnaround time. Furthermore, short-read sequencing inevitably results in gaps in the assembly, and the gaps present in short read-only assemblies are a concern as genes present within that gap may be missed, and assemblies on the edge of a contig (next to the gap) may be of lower quality than assemblies in the middle of the contig. 

The recent development of long-read sequencing technologies such as the Oxford Nanopore (ONP) MinION can potentially provide stand-alone long-read sequencing data with a rapid turnaround time. However, these technologies are still considered error prone despite continuous improvement. That being said, these technologies can bolster short-read analysis with long reads for hybrid analysis [3,4,5,6]. The portability, small footprint, and real-time sequencing capacity of the ONP MinION platforms makes them attractive for clinical use; however, as an emerging technology, work still needs to be performed establishing its usability in a clinical environment [6,7].

Some recent studies have undertaken comparisons of long- and short-read sequencing. Long-read assemblies were generally found to provide more complete assemblies and longer contigs than short-read assemblies, short-read assemblies were more precise than long-read assemblies, and hybrid assemblies were the most complete and accurate of all assemblies overall. In a study investigating the presence of antimicrobial resistance genes (ARGs), it was noted that stand-alone long-read sequencing resulted in occasional false negatives regarding the presence of certain ARGs [8].

*Brucella melitensis* is a key zoonotic bacterial species that is a driver of brucellosis infections, including in the Middle East [9,10,11,12,13,14,15,16]. Brucellosis is an under-diagnosed systemic infection; it is estimated that approximately 90% of human cases go undiagnosed [17,18]. Diagnosis is notably difficult due to lack of specific symptoms, and common testing methodologies vary in sensitivity [17]. If inadequately treated, the infection can progress to long-term, debilitating disease [19]. The gold standard of diagnosis is based on blood culture, but further handling the organism requires strict safety conditions and thus characterization of isolates (e.g., for identifying virulence genes or for performing epidemiological typing) is rarely performed outside reference laboratories. Moreover, the organism is commonly isolated from affected animals during field sampling. As such, *Brucella* spp. are an ideal target for diagnostic genomic sequencing, especially field-deployable long-read sequencing methodologies, to speed diagnosis as well as infer transmission pathways. For example, a case study was reported where a patient with neurobrucellosis was diagnosed by whole-genome metagenomic sequencing (Illumina HiSeq platform) after testing negative on a *Brucella* ELISA IgM [15]; and in another case study, brucellosis was rapidly identified via ONP long-read sequencing. Illumina sequencing has also been utilized in brucellosis outbreak investigations in Israel [11] as well as genomic epidemiology studies [14]. While the MinION has been used to investigate the presence of viral diseases including ebola, rabies, and dengue [20,21], research into sequencing of *Brucella* spp. including with the MinION is limited [22,23] and the application of long-read sequencing on human *B. melitensis* isolates powered by hybrid assemblies has not been attempted. This study aims to investigate long-read sequencing of clinical *B. melitensis*. isolates, and in particular, to compare short-read, hybrid assembly, and long-read sequencing in order to recommend practicable workflow for future use.

## 2. Materials and Methods

### 2.1. Isolate Collection, DNA Extraction, and Sequencing

Eighteen *B. melitensis* isolates from brucellosis cases recovered in blood culture from patients treated at the Soroka University Medical Center, Beer Sheva, Southern Israel were retrieved from the National Brucellosis Reference Laboratory (Kimron Veterinary Institute, Beit Dagan, Israel). Isolates were a convenience sample sub-selected from a larger pool of clinical brucellosis isolates based on available DNA of sufficient quantity (500–1000 ng of total DNA) and quality (A260/A280 ratio of approximately 1.8) for Oxford Nanopore (ONP, Oxford, UK) sequencing. Isolates were extracted using the Qiagen Blood and Tissue kit (Qiagen, Hilden, Germany). Extracted DNA was measured with the QuBit (ThermoFisher, Waltham, MA, USA) and the NanoDrop (ThermoFisher, Waltham, MA, USA) devices to quantify DNA quantity and quality. Fragment length was assessed via BioAnalyzer (Agilent technologies, Santa Clara, CA, USA). DNA was sequenced using Illumina MiSeq platforms (Illumina, San Diego, CA, USA) and the ONP MinION (Oxford Nanopore, Oxford, UK). Culturing, DNA extraction, and Illumina sequencing are described in further detail in a previous publication [16]. For Illumina sequencing, DNA was sequenced using a Miseq V2-500 cycle kit to generate 2 × 250 paired-end reads. For ONP sequencing, a R9.4.1 Flow cell (FLO-MIN106) was used and Ligation Sequencing Kit (SQK-LSK109) was used with some modifications to the ONP protocol. Briefly, during library preparation, the AMPure beads (Beckman Coulter, Pasadena, CA, USA) were washed with 75% ethanol rather than 70% and incubated for 15 min on a rotational mixer during elution of DNA at the end of library preparation as well as the end of adapter ligation and clean-up. Short- and long-read genome assemblies described below are deposited under BioProject number PRJEB50430.

### 2.2. Short Reads Assembly

Short reads from Illumina sequencing underwent quality control (QC, using FastQC, v0.11.5) [24]; and Kraken2, 2.0.7-beta) [25], trimming (using Trimmomatic, v0.39 [26]) and assembly (using SPADes, v3.14.0 [27]; and Pilon, v1.23, [28]) through the INNUca pipeline (v4.2.2) [29]. Default parameters were used.

### 2.3. Long Reads Assembly

Long reads from ONP sequencing were basecalled and demultiplexed (if required) using Guppy (v6.0.1, HAC mode, with config file DNA_r9.4.1_450bps_hac.cfg and default parameters, Oxford Nanopore Technologies, Oxford, UK), and then underwent QC using pycoqc (v2.5.2) [30]. Adapter sequences were removed using porechop (v0.2.4) [31] and reads shorter than 1000 bases were also removed using filtlong (v0.2.1) [32]. The remaining long reads were assembled using dragonflye (v1.0.7), with flye (v2.9-b1768) as the assembler and medaka (v1.5.0, model r941_min_hac_g507, for 4 rounds) as the polisher as described in Wick and Holt (2020) [33]. Default parameters were used unless otherwise noted.

### 2.4. Hybrid Assembly

Short reads and long reads that passed QC and filtering (as mentioned above) underwent hybrid (ONP long reads scaffolded on Illumina short reads) assembly first using the Trycycler workflow (v0.5.0) [34] as described in [35,36]. In brief, the long reads were assembled in trycycler using 15 different assembly attempts (i.e., 5 assemblies from 3 different assemblers in dragonflye (v1.0.7) [37], namely flye (v2.9-b1768) [38], raven (v1.7.0) [39] and miniasm (v0.3-r179) [40]). The trycycler consensus long reads assembly was then polished with 4 rounds of medaka (v1.5.0, model r941_min_hac_g507) [41]. Short reads were then used to polish further using one round of polypolish (v0.4.3) [42] and 2 rounds of POLCA (from MaSuRCA v4.0.4) [43]. Default parameters were used unless otherwise noted.

### 2.5. Downstream Analyses

All genome assemblies were validated as being *B. melitensis* using Kraken2, mlst (v2.18.1) [25], with the pubMLST *Brucella* scheme, Feb2020) [44], QUAST (v5.0.2) [45], BUSCO (v3.0.2) [46], and seqkit (v0.14.0) [47]. Assemblies were annotated for the gaps analysis using prokka (v1.14.5) [48]. The short-read and long-read genome assemblies were compared to the hybrid genome assembly for each isolate separately using NucDiff (v2.0.3) [49], to identify the regions (including genes) missing in either the short-read or long-read assembly. The assembly graphs of the genome assemblies were visually compared using bandage (v0.8.1) [50]. To compare assembly quality, NG50 and NG75 statistics (length of the shortest contig at 50% and 75% of the total reference genome length) and BUSCO statistics were utilized [46,51]. Statistics were carried out in R (Version 4.0.2) with the psych and ggplot2 packages. A total of 51 *B. melitensis* virulence genes were searched for in the genome assemblies using ABRIcate (v1.0.0 [52] with VFDB (2 February 2022) [53] using the parameters ‘--minid 85-mincov 80’, while an additional 10 virulence genes of interest (*BetB*, *BPE275*, *BSPB*, *manA*, *mviN*, *omp19*, *perA*, *PrpA*, *VceC*, and *ureI*) were identified using in silico PCR as previously described [16].

Ad hoc core genome MLST (cgMLST) analysis was conducted with chewBBACA (v2.6.0 [52]; using the BM 16 M complete genome for training Prodigal). Two cgMLST analyses were performed using this method: one using the short-read assemblies, long-read assemblies, and hybrid assemblies of all isolates in order to compare variation in cluster assignment between assembly methods, and one using long-read assemblies only. Minimum spanning trees (MST) were generated and visualized using GrapeTree (v1.5.0, with the MSTreeV2 method) [53]. For single-nucleotide polymorphism (SNP) analysis, all genome assemblies were mapped to the *B. melitensis* reference strain 16M complete genome (accession: GCF_000007125.1) using Snippy (v4.6.0) (accessed on 1 February 2022) (using default parameters and the ‘—ctgs’ input parameter) [54]. Core genome SNPs were then determined using snippy core and recombination sites were masked using Gubbins (v3.0.0) [55]. An MST was generated (using the MSTreeV2 method) and visualized from the final masked cgSNPs alignment (consisting of 3792 core SNPs) with GrapeTree (v1.5.0).

## 3. Results

### 3.1. Read and Assembly Statistics 

Regarding read statistics, the mean read length of long-read sequences ranged from 3393.2 to 8420.8 bp, and mean read length of short-read sequences ranged from 112.7 to177 bp. Q20% (percent of reads with a quality score above 20) and Q30% (percent of reads with a quality score above 30) were higher for short-read sequences than long-read sequences. Regarding Q20%, the median for long reads was 60.65% (range: 48.3–63.75%) and the median for short reads was 93.7% (range: 84.4–96.4%). Regarding Q30%, the median for long reads was 15.8% (range: 9.8–17.7%) and the median for short reads was 90.9% (78.9–94.6%).

**Figure 1:**
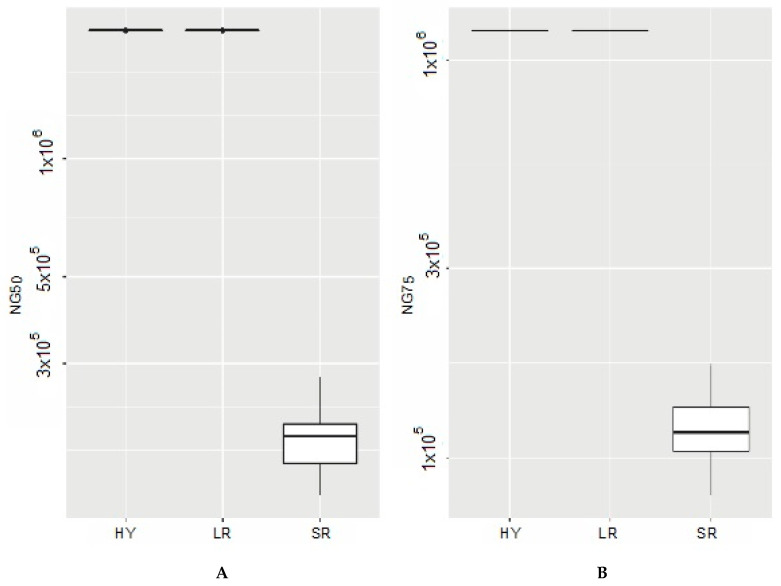
NG50 (**A**) and NG75 (**B**) values for hybrid assembly (HY), long-read assembly (LR), and short-read assembly (SR) of 18 clinical *Brucella melitensis* isolates. NG50 and NG75 are the length of the shortest contig at 50% and 75% of the total reference genome length, respectively.

**Figure 2:**
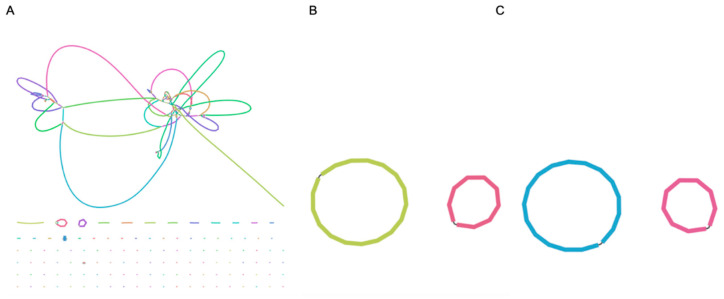
Assembly (bandage) plots of short-read assembly (**A**), long-read assembly (**B**), and hybrid assembly (**C**) for isolate B4 as an exemplar. This figure represents how a complete long-read assembly, visualized as two complete circles with two contigs, can scaffold a fragmented short-read assembly, visualized as multiple fragmented contigs.

Regarding assembly statistics, the median number of contigs was 2 contigs for long-read and hybrid assemblies (range: 2–4 contigs for long-read assemblies, and all hybrid assemblies had 2 contigs) and 38.5 for short-read assemblies (range: 30–60 contigs). NG50 and NG75 values were higher for long-read and hybrid assemblies than short-read assemblies (Figure 1, Table 1). After completion of BUSCO analysis, short-read assemblies had a slightly higher completeness than long-read assemblies. Hybrid assemblies had the same BUSCO values as short-read assemblies. The median largest contig was shorter for short-read assemblies than long-read and hybrid assemblies. Regarding total length and depth, this was fairly similar among assembly types. All descriptive statistics are detailed in Table 1. Regarding scaffolding and the utility of hybrid assembly, Figure 2 represents how a long-read assembly with two contigs can scaffold a fragmented short-read assembly.

### 3.2. Virulence Gene Identification

In total, 51 virulence genes from the VFDB were identified in all assemblies. Results from the in silico PCR for 10 additional virulence genes (*BetB*, *BPE275*, *BSPB*, *manA*, *mviN*, *omp19*, *perA*, *PrpA*, *VceC*, and *ureI*) were also concurrent; all ten virulence genes were identified in short-read, long-read and hybrid assemblies of all isolates. Sequencing errors in long-read assemblies (deletions, substitutions, etc.) were noted for one isolate in *perA*, *omp19*, and *VceC* and for four isolates in *BPE275*. Of the 8 total errors, 5 (62.5%) of the errors were substitutions, 3 (37.5%) were insertions, and none were deletions. In all of these instances, the error was corrected upon hybrid assembly and did not interfere with the identification of the virulence gene in long read-only assemblies. One true variant (present in short-read, long-read, and hybrid assemblies) was noted in one isolate for the gene *BPE275* (C208T). Of note, in one isolate, a virulence gene was initially missed upon hybrid assembly as the full assembly is circular and initially the virulence gene searching tool missed it due to its limitations. Upon closer inspection, the gene was identified in the hybrid assembly.

### 3.3. Phylogeny Comparison

**Figure 3:**
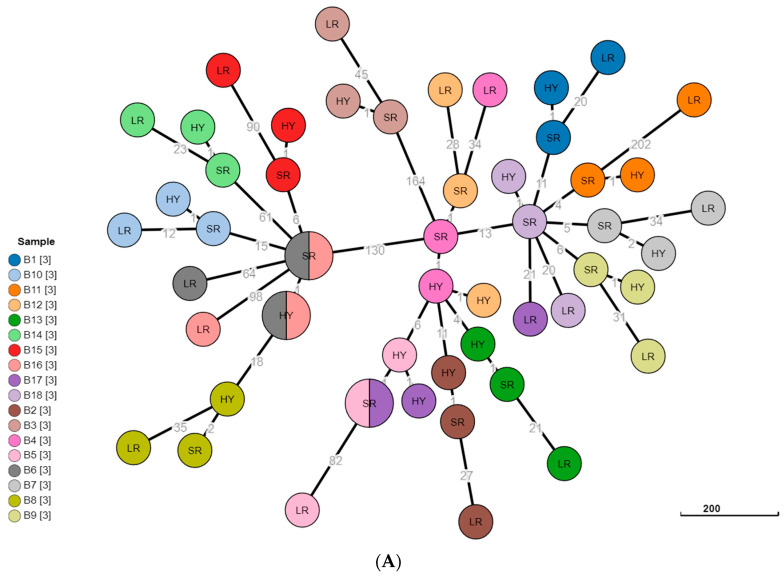

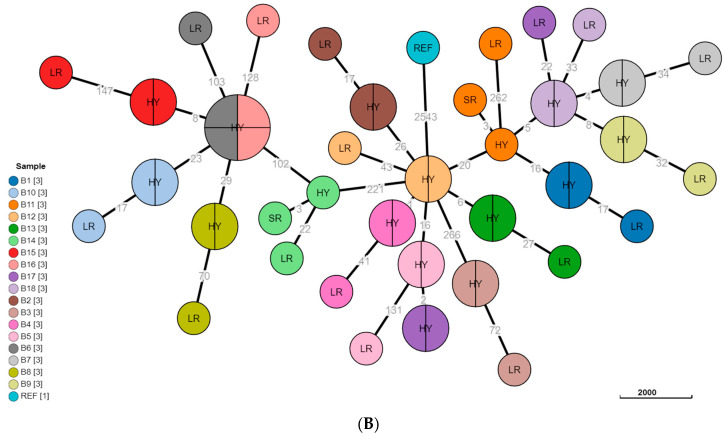
Minimum spanning tree of ad hoc cgMLST (**Panel A**) and SNP (**Panel B**) of 18 clinical *Brucella melitensis* isolates. The tree includes isolates sequenced via long-read (LR) Oxford Nanopore technology, isolates sequenced via short-read (SR) Illumina technology, and hybrid assembly of SRs scaffolded onto LRs. Numbers in grey denote number of allelic differences (**Panel A**) or number of differing single-nucleotide polymorphisms (SNPs) (**Panel B**) between assemblies or isolates, and the nodes are colored according to the isolate number. Nodes with more than one color denote two assemblies of two apparently related isolates that did not have any allelic differences (**Panel A**) or differing SNPs (**Panel B**). In (**Panel B)**, iolates with no differences between the HY and SR assemblies are the same color node with a black line down the middle.

Prior work investigating outbreaks and regional clustering of B. melitensis has noted that up to six allelic differences would be considered an acceptable threshold to consider clustered isolates on a gene-by-gene phylogenetic analysis (cgMLST) as epidemiologically related [56]. Studies in our region [13,14] and our cumulative experience in local investigations of brucellosis (unpublished data) suggest up to 10–15 differing alleles or SNPs may still constitute a practicable threshold for relatedness. As seen in Figure 3, most sequenced isolates tend to cluster together according to short-read, long-read, and hybrid assemblies. However, in almost all cases, the long-read assemblies exhibited a much higher allelic difference from the hybrid assembly than the short-read assembly (Figure 3 Panel A). A similar finding was noted when the number of differing single-nucleotide polymorphisms (SNPs) was compared between long-read assemblies and hybrid assemblies and between short-read assemblies and hybrid assemblies (Figure 3 Panel B). For all clusters, the number of differing alleles between long-read and hybrid assemblies ranged between 13 and 203 allelic differences and 17 and 285 differing SNPs (median: 35.5 allelic differences, 42 differing SNPs). In comparison, the number of allelic differences between short-read and hybrid assemblies ranged between 1 and 3 allelic differences and 0 and 3 differing SNPs (median: 1 allelic difference, 0 differing SNPs). In 17 out of 18 isolates (94.4%), the number of differing alleles between long-read and hybrid assemblies was above the relaxed threshold (15 alleles) that would define epidemiological relatedness. Only one long-read/short-read/hybrid cluster (B10) fit within the parameters regarding less than 15 allelic differences (Figure 3, Panel A). Of note, the long-read assemblies of isolate B17 did not cluster with the short-read and hybrid counterparts at all; this mis-assignment is likely due to overall poor quality of long-read sequence in that sample (See: Q20% and Q30% results).

**Figure 4:**
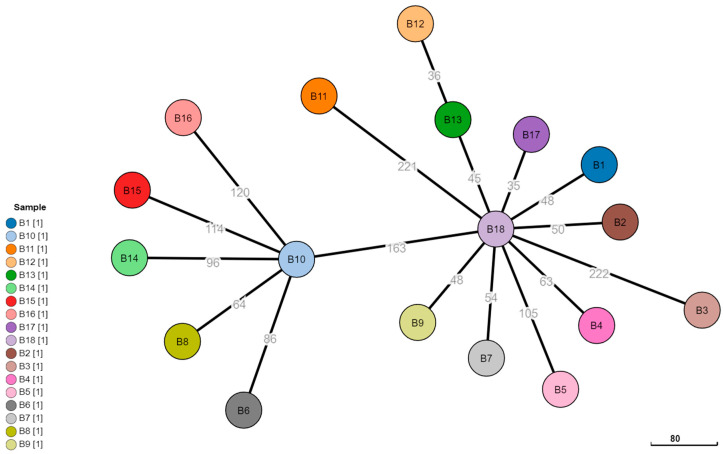
Minimum spanning tree of ad hoc cgMLST long-read assemblies of 18 clinical *Brucella melitensis* isolates. Numbers in grey denote number of allelic differences between the isolates, and the nodes are colored according to the isolate number.

When a phylogenetic tree was constructed, only with long-read assemblies (Figure 4), all isolates exhibited allelic differences that far exceed the epidemiological relatedness threshold and in a scenario using only ONP sequencing, no clear chains of transmission or relatedness between cases would have been evident. Moreover, short-read and hybrid assemblies showed several clear case clusters such as B6-B16 and B4-B12-B13, but these clusters were not evident in the long-read assemblies.

### 3.4. What Is in the Gaps?

Analysis of the gaps present in short-read assemblies was conducted to assess what would have been missed if short-read assembly was used alone vs. in hybrid assembly. Genes were identified in the gaps of all 18 short-read assemblies (median: 10 genes, range: 9–17 genes). The majority of the genes missed by short read-only assembly are transposable elements (e.g., transposases of the IS3, IS5, and IS6 families) and do not, to the author’s knowledge, have clinical relevance. Furthermore, repetitive regions such as these are known to be poorly sequenced by short-read sequencing methodologies, so missing these genes in short-read gaps is expected [57].

## 4. Discussion

### 4.1. Study Summary

This study aimed to investigate long-read sequencing of clinical *B. melitensis* isolates, and in particular, to compare short read-based, long read-based and hybrid assemblies in order to recommend practicable workflow for future use. Overall, virulence genes were consistently identified in short-read, hybrid, and long-read assemblies. While there were instances of gaps in the short-read assembly and errors in long-read assemblies, this did not ultimately affect identification of virulence genes. These findings are similar to work investigating Illumina vs. ONP sequencing with *Escherichia coli* surrogate strain isolates [58]. In general, long-read sequencing had notable limitations in regard to phylogenetic analysis, and long-read assemblies generally failed to cluster closely enough with their short-read and hybrid counterparts to be considered as epidemiologically related based on a previously established threshold. One long-read assembly clustered with an entirely different isolate cluster; however, this has been observed in previous studies focusing on other bacterial genera, and as also seen in the research, this was reported to be corrected upon hybrid assembly [4,8,59]. Furthermore, when a tree was generated with long-read assemblies alone, related isolates that clustered together from short-read or hybrid assemblies no longer clustered together. The superiority of short-read assemblies to long-read assemblies in regard to phylogenetic resolution has also been observed in a benchmarking study of *Salmonella* isolates [8]. Ultimately, complete genomes resulting from hybrid assembly will allow for more confident analysis of genomes using a gene-by-gene approach as well as SNP-level analysis. Depth and BUSCO completeness percentages for short-read and long-read assemblies were similar, which disagrees with other studies [60], but it again bears mentioning that *B. melitensis* may be less complex to analyze than other studied organisms.

### 4.2. Implications for Field-Deployed or Low-Resource Settings

The ONP platform is popular due to its utility as a field-deployable sequencing platform; furthermore, in low-resource settings, it can provide whole genomes faster and with fewer resources. The MinION has been noted in multiple papers to be highly useful in backcountry or low-resource settings, including tent-based or car-based research efforts [20,61,62]. For infectious diseases such as brucellosis, epidemiological trace-back is often critical in the face of outbreaks, cases associated with international travel or even cases in non-endemic regions. Whole-genome sequencing can also be used in this regard; for example, a study utilizing Illumina sequencing in Germany noted that a large number of brucellosis cases were of Middle Eastern origin [63]. Previous work has noted that the ONP platform can provide speedy diagnosis of brucellosis; for example, when Gündoğdu et al.’s (2019) ONP usage for clinical diagnosis identified the first read for *B. melitensis* in 30 min [23]. 

Given the virulence gene findings of this study, it is apparent that long read-only assemblies can provide actionable data regarding *Brucella* spp. virulence gene presence. However, the findings of the phylogenetic comparisons warrant further study; while the long-read sequencing produced good resolution in this regard, differences were observed among the assembly types as far as number of allelic differences or SNPs was concerned. It is possible typing using long reads could be improved with longer sequencing times (which necessitate faster consumption of costly flow cells). As the genome of *B. melitensis* is highly conserved and isolates from the same region, including in the Israeli Negev desert, tightly cluster together, high resolution for accurate phylogenetic analysis is very important for this particular organism in regard to epidemiological or outbreak investigations [13,14]. For the investigation of specific genes in other clinically-relevant organisms, more research is needed, as while this study found that the ONP platform was consistent in identifying virulence genes, recent studies have noted that ONP technology had inconsistent performance regarding the detection of antimicrobial resistance genes in Gram-negative bacteria when compared to Illumina technology [4,9,59]. Overall, the findings of this study suggest that in operational settings where information is needed on the virulence genes of *B. melitensis*, stand-alone long-read sequencing provides comparable data to short-read sequencing in a shorter amount of time. Regarding the utility for epidemiological and outbreak settings, there is a need for further refinement and validation of the method, perhaps via longer sequencing time.

### 4.3. Limitations and Future Research

The primary limitations of this study were the small number of isolates and lower quality of long-read sequences and lower depth for some of the samples. Future research optimizing ONP sequencing for the epidemiological investigation of *Brucella* spp. is also needed, especially regarding field-based sequencing in endemic areas. Other research investigating ONP sequencing has noted this need for future research before stand-alone long-read sequencing is utilized in the clinical environment [4]. Concordantly, future research should be undertaken to “downsample” long-read platforms to determine at what point depth suffers and falls significantly below short-read methodologies. Furthermore, future research should be undertaken using concordant blood, cerebral spinal fluid, or other relevant clinical materials to investigate the utility of ONP and hybrid sequencing for culture-independent *B. melitensis* diagnosis.

## 5. Conclusions

This study aimed to investigate long-read sequencing and hybrid sequencing of clinical *B. melitensis* isolates, with the specific intention to compare short read-based, long read-based and hybrid assemblies in order to recommend practicable workflow for future clinical use. Overall, it is key to note that all virulence genes were identified in all isolates using all sequencing and assembly methodologies; however, caution is warranted upon hybrid assembly. For phylogeny, some differences and inconsistencies in clustering were observed for the short-read and long-read assemblies; therefore, further research is needed regarding these technologies for phylogenomic research of *Brucella* spp.

## Figures and Tables

**Table 1:** Assembly statistics of hybrid assembly, long-read assemblies (Oxford Nanopore platform), and short-read assemblies (Illumina platform) of 18 clinical Brucella melitensis isolates.

**Hybrid Assemblies**				
	**Mean**	**Median**	**Minimum**	**Maximum**
Total length (bp)	3.31 × 10^6^	3.31 × 10^6^	3.31 × 10^6^	3.31 × 10^6^
Largest contig (bp)	2.13 × 10^6^	2.13 × 10^6^	2.13 × 10^6^	2.13 × 10^6^
NG50 (bp)	2.13 × 10^6^	2.13 × 10^6^	2.13 × 10^6^	2.13 × 10^6^
NG75 (bp)	1.19 × 10^6^	1.19E × 10^6^	1.19 × 10^6^	1.19 × 10^6^
Average coverage depth (x)	115.7	110.0	42.0	177.0
BUSCO completeness (%)	97.9	98.0	97.3	98.0
**Long-Read Assemblies**				
	**Mean**	**Median**	**Minimum**	**Maximum**
Total length (bp)	3.32 × 10^6^	3.32 × 10^6^	3.31 × 10^6^	3.32 × 10^6^
Largest contig (bp)	2.13 × 10^6^	2.13 × 10^6^	2.13 × 10^6^	2.13 × 10^6^
NG50 (bp)	2.13 × 10^6^	2.13 × 10^6^	2.13 × 10^6^	2.13 × 10^6^
NG75 (bp)	1.19 × 10^6^	1.19 × 10^6^	1.19 × 10^6^	1.19 × 10^6^
Average coverage depth (x)	115.5	110.0	42.0	176.0
BUSCO completeness (%)	97.4	98.0	93.9	98.0
**Short-Read Assemblies**				
	**Mean**	**Median**	**Minimum**	**Maximum**
Total length (bp)	3.31 × 10^6^	3.30 × 10^6^	3.29 × 10^6^	3.47 × 10^6^
Largest contig (bp)	4.32 × 10^5^	4.17 × 10^5^	3.59 × 10^5^	6.10 × 10^5^
NG50 (bp)	1.95 × 10^5^	1.95 × 10^5^	1.38 × 10^5^	2.76 × 10^5^
NG75 (bp)	1.18 × 10^5^	1.16 × 10^5^	8.06 × 10^4^	1.72 × 10^5^
Average coverage depth (x)	119.6	116.5	45.0	185.0
BUSCO completeness (%)	97.9	98.0	97.3	98.0

## Data Availability

Assemblies are available online at BioProject number PRJEB50430.

## References

[1] Bachmann N.L., Rockett R.J., Timms V.J., Sintchenko V. (2018). Advances in clinical sample preparation for identification and characterization of bacterial pathogens using metagenomics. Front. Public Health.

[2] Gardy J.L., Loman N.J. (2018). Towards a genomics-informed, real-time, global pathogen surveillance system. Nat. Rev. Genet..

[3] De Maio N., Shaw L.P., Hubbard A., George S., Sanderson N.D., Swann J., Wick R., AbuOun M., Stubberfield E., Hoosdally S.J. (2019). Comparison of long-read sequencing technologies in the hybrid assembly of complex bacterial genomes. Microb. Genom..

[4] Magi A., Semeraro R., Mingrino A., Giusti B., D’Aurizio R. (2018). Nanopore sequencing data analysis: State of the art, applications and challenges. Brief. Bioinform..

[5] Tyler A.D., Mataseje L., Urfano C.J., Schmidt L., Antonation K.S., Mulvey M.R., Corbett C.R. (2018). Evaluation of Oxford Nanopore’s MinION Sequencing Device for Microbial Whole Genome Sequencing Applications. Sci. Rep..

[6] Susilawati T.N., Jex A.R., Cantacessi C., Pearson M., Navarro S., Susianto A., Loukas A.C., McBride W.J.H. (2016). Deep sequencing approach for investigating infectious agents causing fever. Eur. J. Clin. Microbiol. Infect. Dis..

[7] Yu X., Jiang W., Shi Y., Ye H., Lin J. (2019). Applications of sequencing technology in clinical microbial infection. J. Cell Mol. Med..

[8] Chen Z., Kuang D., Xu X., González-Escalona N., Erickson D.L., Brown E., Meng J. (2020). Genomic Analyses of Multidrug-Resistant Salmonella Indiana, Typhimurium, and Enteritidis Isolates Using MinION and MiSeq Sequencing Technologies. PLoS ONE.

[9] Alghoribi M.F., Zidan K.H., Alswaji A.A., Alhafufi A.N., Ahmed A., Balkhy H.H. (2018). Whole-Genome Sequence of a Brucella melitensis Strain Isolated from Sheep in Saudi Arabia. Microbiol. Resour. Announc..

[10] Aljanazreh B., Alzatari K., Tamimi A., Alsaafeen M.H., Hassouneh W., Ashhab Y. (2021). Brucellosis re-emergence after a decade of quiescence in Palestine, 2015–2017: A seroprevalence and molecular characterization study. Transbound. Emerg. Dis..

[11] Anis E., Leventhal A., Grotto I., Gandacu D., Warshavsky B., Shimshony A., Israeli A. (2011). Recent trends in human brucellosis in Israel. Isr. Med Assoc. J. IMAJ.

[12] Rabinowitz P., Zilberman B., Motro Y., Roberts M.C., Greninger A., Nesher L., Ben-Shimol S., Yagel Y., Gdalevich M., Sagi O. (2021). Whole Genome Sequence Analysis of Brucella melitensis Phylogeny and Virulence Factors. Microbiol. Res..

[13] Bardenstein S., Gibbs R.E., Yagel Y., Motro Y., Moran-Gilad J. (2021). Brucellosis Outbreak Traced to Commercially Sold Camel Milk through Whole-Genome Sequencing, Israel. Emerg. Infect. Dis..

[14] Zilberman B., Motro Y., Sagi O., Kornspan D., Ben-Shimol S., Gdalevich M., Yagel Y., Davidovitch N., Khalfin B., Rabinowitz P. (2022). Genomic Epidemiology of Clinical Brucella Melitensis Isolates from Southern Israel. Microorganisms.

[15] Vered O., Simon-Tuval T., Yagupsky P., Malul M., Cicurel A., Davidovitch N. (2015). The Price of a Neglected Zoonosis: Case-Control Study to Estimate Healthcare Utilization Costs of Human Brucellosis. PLoS ONE.

[16] Mongkolrattanothai K., Naccache S.N., Bender J.M., Samayoa E., Pham E., Yu G., Dien Bard J., Miller S., Aldrovandi G., Chiu C.Y. (2017). Neurobrucellosis: Unexpected Answer From Metagenomic Next-Generation Sequencing. J. Pediatr. Infect. Dis. Soc..

[17] Avijgan M., Rostamnezhad M., Jahanbani-Ardakani H. (2019). Clinical and Serological Approach to Patients with Brucellosis: A Common Diagnostic Dilemma and a Worldwide Perspective. Microb. Pathog..

[18] Mantur B.G., Amarnath S.K., Shinde R.S. (2007). Review of Clinical and Laboratory Features of Human Brucellosis. Indian J. Med. Microbiol..

[19] Al Dahouk S., Nöckler K. (2011). Implications of Laboratory Diagnosis on Brucellosis Therapy. Expert Rev. Anti Infect. Ther..

[20] Brunker K., Marston D.A., Horton D.L., Cleaveland S., Fooks A.R., Kazwala R., Ngeleja C., Lembo T., Sambo M., Mtema Z.J. (2015). Elucidating the phylodynamics of endemic rabies virus in eastern Africa using whole-genome sequencing. Virus Evol..

[21] Greninger A.L., Naccache S.N., Federman S., Yu G., Mbala P., Bres V., Stryke D., Bouquet J., Somasekar S., Linnen J.M. (2015). Rapid metagenomic identification of viral pathogens in clinical samples by real-time nanopore sequencing analysis. Genome Med..

[22] Bolotin V., Kovalenko G., Marchenko N., Solodiankin O., Rudova N., Kutsenko V., Bortz E., Gerilovych A., Drown D.M. (2021). Complete Genome Sequence of Brucella abortus 68, Isolated from Aborted Fetal Sheep in Ukraine. Microbiol. Resour. Announc..

[23] Gündoğdu A., Ulu-Kilic A., Kilic H., Nalbantoglu O.U. (2019). Rapid detection of difficult-to-culture bacterial pathogens using real-time nanopore sequencing. Infect. Dis. Clin. Microbiol..

[24] Babraham Bioinformatics (2021). FastQC. A Quality Control tool for High Throughput Sequence Data. https://www.bioinformatics.babraham.ac.uk/projects/fastqc/.

[25] Wood D.E., Lu J., Langmead B. (2019). Improved metagenomic analysis with Kraken 2. Genome Biol..

[26] Bolger A.M., Lohse M., Usadel B. (2014). Trimmomatic: A flexible trimmer for Illumina sequence data. Bioinformatics.

[27] Prjibelski A., Antipov D., Meleshko D., Lapidus A., Korobeynikov A. (2020). Using SPAdes De Novo Assembler. Curr. Protoc. Bioinforma..

[28] Walker B.J., Abeel T., Shea T., Priest M., Abouelliel A., Sakthikumar S., Cuomo C.A., Zeng Q., Wortman J., Young S.K. (2014). Pilon: An Integrated Tool for Comprehensive Microbial Variant Detection and Genome Assembly Improvement. PLoS ONE.

[29] INNUca.py (2021). Bioinformatics @ Molecular Microbiology and Infection Unit. https://github.com/B-UMMI/INNUca.

[30] Leger A., Leonardi T. (2019). pycoQC, interactive quality control for Oxford Nanopore Sequencing. J. Open Source Softw..

[31] Wick R. (2021). Porechop. https://github.com/rrwick/Porechop.

[32] Wick R. (2021). rrwick/Filtlong. https://github.com/rrwick/Filtlong.

[33] Wick R.R., Holt K.E. (2020). Benchmarking of long-read assemblers for prokaryote whole genome sequencing. F1000Research.

[34] Wick R.R., Judd L.M., Cerdeira L.T., Hawkey J., Méric G., Vezina B., Wyres K.L., Holt K.E. (2021). Trycycler: Consensus long-read assemblies for bacterial genomes. Genome Biol..

[35] Wick R. Trycycler. GitHub. https://github.com/rrwick/Trycycler.

[36] Wick R.R. (2021). Trycycler. Zenodo. https://zenodo.org/record/5769082.

[37] Petit R.A. (2022). Dragonflye. https://github.com/rpetit3/dragonflye.

[38] Kolmogorov M., Yuan J., Lin Y., Pevzner P.A. (2019). Assembly of Long, Error-Prone Reads Using Repeat Graphs. Nat. Biotechnol..

[39] Vaser R., Šikić M. (2021). Time- and Memory-Efficient Genome Assembly with Raven. Nat. Comput. Sci..

[40] Oxford Nanopore Technologies (2022). Medaka. https://github.com/nanoporetech/medaka.

[41] Li H. (2021). lh3/miniasm. https://github.com/lh3/miniasm.

[42] Wick R.R., Holt K.E. (2022). Polypolish: Short-Read Polishing of Long-Read Bacterial Genome Assemblies. PLoS Comput. Biol..

[43] Zimin A.V., Marçais G., Puiu D., Roberts M., Salzberg S.L., Yorke J.A. (2013). The MaSuRCA Genome Assembler. Bioinformatics.

[44] Seemann T. (2021). mlst. https://github.com/tseemann/mlst.

[45] Mikheenko A., Prjibelski A., Saveliev V., Antipov D., Gurevich A. (2018). Versatile genome assembly evaluation with QUAST-LG. Bioinformatics.

[46] Manni M., Berkeley M.R., Seppey M., Simao F.A., Zdobnov E.M. (2021). BUSCO Update: Novel and Streamlined Workflows along with Broader and Deeper Phylogenetic Coverage for Scoring of Eukaryotic, Prokaryotic, and Viral Genomes. http://arxiv.org/abs/2106.11799.

[47] Shen W., Le S., Li Y., Hu F. (2016). SeqKit: A Cross-Platform and Ultrafast Toolkit for FASTA/Q File Manipulation. PLoS ONE.

[48] Seemann T. (2014). Prokka: Rapid prokaryotic genome annotation. Bioinformatics.

[49] Khelik K., Lagesen K., Sandve G.K., Rognes T., Nederbragt A.J. (2017). NucDiff: In-depth characterization and annotation of differences between two sets of DNA sequences. BMC Bioinform..

[50] Wick R.R., Schultz M.B., Zobel J., Holt K.E. (2015). Bandage: Interactive visualization of de novo genome assemblies. Bioinformatics.

[51] Bradnam K.R., Fass J.N., Alexandrov A., Baranay P., Bechner M., Birol I., Boisvert S., Chapman J.A., Chapuis G., Chikhi R. (2013). Assemblathon 2: Evaluating de novo methods of genome assembly in three vertebrate species. GigaScience.

[52] Silva M., Machado M.P., Silva D.N., Rossi M., Moran-Gilad J., Santos S., Ramirez M., Carrico J.A. (2018). chewBBACA: A complete suite for gene-by-gene schema creation and strain identification. Microb. Genomics.

[53] Zhou Z., Alikhan N.-F., Sergeant M.J., Luhmann N., Vaz C., Francisco A.P., Carriço J.A., Achtman M. (2018). GrapeTree: Visualization of core genomic relationships among 100,000 bacterial pathogens. Genome Res..

[54] Seemann T. (2022). Snippy. https://github.com/tseemann/snippy.

[55] Croucher N.J., Page A.J., Connor T.R., Delaney A.J., Keane J.A., Bentley S.D., Parkhill J., Harris S.R. (2015). Rapid Phylogenetic Analysis of Large Samples of Recombinant Bacterial Whole Genome Sequences Using Gubbins. Nucleic Acids Res..

[56] Janowicz A., De Massis F., Ancora M., Cammà C., Patavino C., Battisti A., Prior K., Harmsen D., Scholz H., Zilli K. (2018). Core Genome Multilocus Sequence Typing and Single Nucleotide Polymorphism Analysis in the Epidemiology of Brucella Melitensis Infections. J. Clin. Microbiol..

[57] Wick R.R., Judd L.M., Gorrie C.L., Holt K.E. (2017). Unicycler: Resolving Bacterial Genome Assemblies from Short and Long Sequencing Reads. PLoS Comput. Biol..

[58] Therrien D.A., Konganti K., Gill J.J., Davis B.W., Hillhouse A.E., Michalik J., Cross H.R., Smith G.C., Taylor T.M., Riggs P.K. (2021). Complete Whole Genome Sequences of Escherichia coli Surrogate Strains and Comparison of Sequence Methods with Application to the Food Industry. Microorganisms.

[59] Chen Z., Erickson D.L., Meng J. (2021). Polishing the Oxford Nanopore long-read assemblies of bacterial pathogens with Illumina short reads to improve genomic analyses. Genomics.

[60] Khezri A., Avershina E., Ahmad R. (2021). Hybrid Assembly Provides Improved Resolution of Plasmids, Antimicrobial Resistance Genes, and Virulence Factors in Escherichia coli and Klebsiella pneumoniae Clinical Isolates. Microorganisms.

[61] Quick J., Loman N.J., Duraffour S., Simpson J.T., Severi E., Cowley L., Bore J.A., Koundouno R., Dudas G., Mikhail A. (2016). Real-time, portable genome sequencing for Ebola surveillance. Nature.

[62] Walter M.C., Zwirglmaier K., Vette P., Holowachuk S.A., Stoecker K., Genzel G.H., Antwerpen M.H. (2017). MinION as part of a biomedical rapidly deployable laboratory. J. Biotechnol..

[63] Georgi E., Walter M.C., Pfalzgraf M.-T., Northoff B.H., Holdt L.M., Scholz H.C., Zoeller L., Zange S., Antwerpen M.H. (2017). Whole genome sequencing of Brucella melitensis isolated from 57 patients in Germany reveals high diversity in strains from Middle East. PLoS ONE.

